# Microsatellite and mtDNA analysis of lake trout, *Salvelinus namaycush*, from Great Bear Lake, Northwest Territories: impacts of historical and contemporary evolutionary forces on Arctic ecosystems

**DOI:** 10.1002/ece3.439

**Published:** 2013-01-10

**Authors:** Les N Harris, Kimberly L Howland, Matthew W Kowalchuk, Robert Bajno, Melissa M Lindsay, Eric B Taylor

**Affiliations:** 1 Fisheries and Oceans Canada501 University Crescent, Winnipeg, MB, Canada, R3T 2N6; 2 Department of Biological Sciences, University of ManitobaWinnipeg, MB, Canada, R3T 2N2; 3 Department of Zoology, Biodiversity Research Centre and Beaty Biodiversity Museum, University of British Columbia6270 University Blvd, Vancouver, BC, Canada, V6T 1Z4

**Keywords:** Gene flow, glaciations, history, panmictic, *Salvelinus*

## Abstract

Resolving the genetic population structure of species inhabiting pristine, high latitude ecosystems can provide novel insights into the post-glacial, evolutionary processes shaping the distribution of contemporary genetic variation. In this study, we assayed genetic variation in lake trout (*Salvelinus namaycush*) from Great Bear Lake (GBL), NT and one population outside of this lake (Sandy Lake, NT) at 11 microsatellite loci and the mtDNA control region (d-loop). Overall, population subdivision was low, but significant (global *F*_ST_ θ = 0.025), and pairwise comparisons indicated that significance was heavily influenced by comparisons between GBL localities and Sandy Lake. Our data indicate that there is no obvious genetic structure among the various basins within GBL (global *F*_ST_ = 0.002) despite the large geographic distances between sampling areas. We found evidence of low levels of contemporary gene flow among arms within GBL, but not between Sandy Lake and GBL. Coalescent analyses suggested that some historical gene flow occurred among arms within GBL and between GBL and Sandy Lake. It appears, therefore, that contemporary (ongoing dispersal and gene flow) and historical (historical gene flow and large founding and present-day effective population sizes) factors contribute to the lack of neutral genetic structure in GBL. Overall, our results illustrate the importance of history (e.g., post-glacial colonization) and contemporary dispersal ecology in shaping genetic population structure of Arctic faunas and provide a better understanding of the evolutionary ecology of long-lived salmonids in pristine, interconnected habitats.

## Introduction

The extent of genetic structure among, and variation within, populations of north temperate faunas is expected to be markedly different from that exhibited by populations from more southerly latitudes, which were exempt from the direct impacts of Pleistocene glaciations (Hewitt 1996). Fishes in particular, given their reliance on aquatic habitats for survival, were clearly impacted by glacial events (e.g., Bernatchez and Wilson 1998; Weider and Hobaek 2000; Wilson and Veraguth 2010). For example, populations with contemporary ranges spanning areas formerly glaciated during the Pleistocene Epoch are expected to show reduced levels of genetic variation and historical effective population size (*N*_E_), as well as reduced levels of DNA sequence and phylogenetic divergence compared with those from unglaciated areas (Hewitt 1996; Bernatchez and Wilson 1998; Hewitt 2004). Differences in patterns between geography and genetic variation are also expected. For instance, decreases in genetic variation with increasing distance from glacial refugia have been observed in several species of fishes (Stamford and Taylor 2004; Harris and Taylor 2010a). Furthermore, isolation and survival in distinct glacial refugia should result in clear phylogeographic groupings of populations (Bernatchez and Dodson 1991; Wilson and Hebert 1998; Stamford and Taylor 2004), although these patterns can be distorted as a result of contemporary secondary contact among refugial groups (Lu et al. 2001; Turgeon and Bernatchez 2001a,b; Adams et al. 2006). Evolutionary processes such as founder events associated with post-glacial dispersal and historical bottlenecks as a result of glacial cycles are offered as potential explanations for some of these observations (Bernatchez et al. 1989; Hewitt 1996; Langefors 2005).

Although current patterns of phylogeography, population structure, and genetic variation among and within populations of northern fishes can be largely explained by historical events, contemporary factors also contribute to these patterns. For example, contemporary connectivity facilitating gene flow among populations will reduce genetic differentiation (Dionne et al. 2008; Shaddick et al. 2011) and can mask some of the historical influences on, or complicate inferences regarding, divergence and population structure (Turgeon and Bernatchez 2001b; Adams et al. 2006). Alternatively, recent reductions in population size, resulting from anthropogenic disturbances (Stamford and Taylor 2005; Hanfling and Weetman 2006), for example, can reduce genetic variation and promote genetic divergence through genetic drift, especially in small populations (Consuegra et al. 2005; Vaha et al. 2007; Whiteley et al. 2010). Furthermore, contemporary landscape and environmental features of aquatic ecosystems (e.g., stream hydrology, salinity, temperature, turbidity), and their influence on connectivity and/or divergent natural selection may influence present-day genetic structuring (Costello et al. 2003; Dionne et al. 2008; Leclerc et al. 2008; Tamkee et al. 2010). Disentangling the relative influences of historical and contemporary factors, such as historical gene flow versus contemporary dispersal, on population genetic structure can present formidable challenges (Sobel et al. 2010; Harris and Taylor 2010b).

One way to increase our understanding of the relative roles of historical and contemporary influences on genetic diversity and population structure is to use comparative methods. The use of multi-locus data of varying evolutionary rates (e.g., nuclear vs. mitochondrial DNA variation) has proven useful in a variety of studies for isolating specific evolutionary processes involved in driving contemporary population structure (Lu et al. 2001; Johnson et al. 2003; Taylor et al. 2008). Microsatellite loci, with estimated rates of mutation of approximately 5 × 10^−3^– 5 × 10^−5^ (Jarne and Lagoda 1996), provide excellent resolution to understand the role that microevolutionary processes such as contemporary gene flow play in influencing the genetic structure of post-glacial populations (Angers and Bernatchez 1998; Koskinen et al. 2002; Gomez-Uchida et al. 2009; Warnock et al. 2010). Alternatively, the mutation rate of mitochondrial DNA (mtDNA) is much lower than that exhibited by microsatellite loci (e.g., 1 × 10^−8^, (Haag-Liautard et al. 2008) and therefore can be informative for capturing signals of historical evolutionary processes influencing genetic structure and speciation (Bernatchez and Dodson 1991; Wilson and Hebert 1998; Wang 2010). Relying solely on one approach may result in misleading conclusions, whereas combining these approaches can prove very useful for disentangling historical versus contemporary influences on population structure and genetic diversity.

Arctic systems provide unique opportunities for investigating the roles of historical and contemporary processes in shaping the genetic structure in populations of freshwater fishes. Virtually all Arctic populations of fishes were impacted by Pleistocene glaciation events through the displacement of populations during glacial maxima and then the recolonization of contemporary ranges when the ice-sheets receded (Lindsey and McPhail 1986; Pielou 1991). Some groups of fishes dispersed remarkable distances across numerous watersheds post-glacially to occupy their current range (Bernatchez and Dodson 1991; Rempel and Smith 1998; Wilson and Hebert 1998; Witt et al. 2011) and these groups provide excellent systems to assess how historical dispersal patterns interact with contemporary dispersal among populations in the evolution of population structure. Furthermore, many Arctic systems are characterized by complex and dynamic watersheds harboring a multitude of populations, many of which exist in a meta-population framework. This permits the study of processes such as contemporary dispersal, gene flow, and source-sink population dynamics (Dias 1996; Palstra et al. 2007) in shaping contemporary population structure. In addition, many of these systems are virtually pristine, providing ample opportunity to study population structure in fishes that have not been anthropogenically impacted. Arctic populations of fishes are relatively less studied than those at more southerly latitudes and therefore the evolutionary ecology of many Arctic fish populations remains unresolved (e.g., Cook et al. 2012).

**Figure 1:**
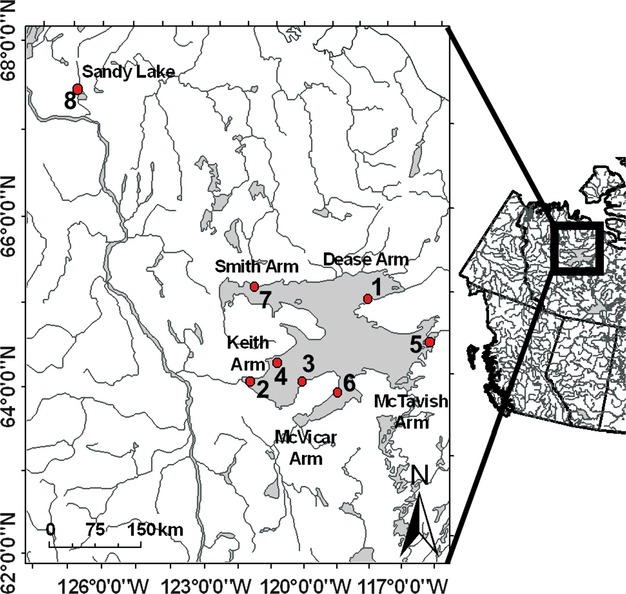
Map of the study area showing sampling locations among arms within Great Bear Lake and in Sandy Lake, Northwest Territories, Canada. Numbers refer to the locations listed in Table 1.

One such Arctic system, Great Bear Lake (GBL, Fig. 1) in Canada's Northwest Territories (NT, Fig. 1) is the largest lake entirely within Canada (Johnson 1975a). Characterized by five distinct basins (from herein referred to as arms), GBL was at one time encompassed by glacial Lake McConnell (Smith 1994). The lake is oligotrophic and is characterized by very low species diversity (Johnson 1975b). Lake trout (*Salvelinus namaycush*) are one of the most abundant and widely distributed fishes in this system, found at all depths and temperatures (Johnson 1975b). The biology and evolutionary ecology of lake trout is relatively well studied in other large lakes, for example, the heavily anthropogenically impacted Laurentian Great Lakes (Moore and Bronte 2001; Guinand et al. 2003; Page et al. 2004; Page 2005; Guinand et al. 2006), but population structure of lake trout in northern systems, including GBL, has not been well studied (but see Northrup et al. 2010). The only molecular study involving GBL lake trout included a small number of fish (*N* = 5) for a mitochondrial DNA analysis of restriction fragment length polymorphism (RFLP) to assess phylogeographic structure and post-glacial dispersal of lake trout throughout its Canadian range (Wilson and Hebert 1998). This study suggested that the majority of lake trout from GBL survived in a Beringian refuge (Wilson and Hebert 1998). Additional recent studies of lake trout in GBL have examined morphological variation and trophic resource polymorphism in this species (Blackie et al. 2003; Alfonso 2004).

To further our understanding regarding the genetic population structure of lake trout from large, pristine lake systems, we assayed microsatellite DNA and mtDNA sequence variation in lake trout from GBL and one lake (Sandy Lake, Fig. 1) from outside of this system. Specifically, we were interested in the geographic scale and extent of population structure in this spatially complex system and in resolving the role of both contemporary ecology (e.g., dispersal and subsequent gene flow) and historical events (survival in and dispersal from, Pleistocene glacial refugia) in shaping this structure. Disentangling the extent to which both of these processes operate to shape contemporary population structure remains an important challenge in evolutionary ecology (Petit and Excoffier 2009). Our study represents one of the few molecular assessments of population structure in lake trout from large, northern post-glacial lakes (but see Northrup et al. 2010).

## Materials and Methods

### Sample collection and molecular methods

Samples of lake trout assayed in this study (*N* = 596) were obtained between 2002 and 2006 from seven locations within GBL. Samples from GBL were collected from all five major arms (Fig. 1) with the exception of Keith Arm, the largest arm in this system, in which three locations were sampled to assess intra-arm variation. Lake trout were not sampled on spawning locations as information on these areas is generally lacking for this system. Initial assessments revealed virtually no population structure within GBL. As such, to assess the power of the loci used in this study to resolve structure that likely exists, we included an additional lake, Sandy Lake, NT (Table 1, Fig. 1) in our analysis. Sandy Lake was chosen because, although it is in the same drainage (the Mackenzie River system) as GBL, it is located some 650 km downstream and is much smaller and thus probably ecologically distinct from GBL. Consequently, we expected that contemporary gene flow between GBL and Sandy Lake would be limited or absent and they should therefore be genetically differentiated from each other. By contrast, GBL and Sandy Lake survived the last glaciation within the same glacial refugium (Beringia) and, therefore, there would likely be some degree of historical gene flow between these populations. All tissue was stored in 95% ethanol or a 20% DMSO / NaCl solution prior to DNA extraction using Qiagen DNeasy tissue extraction kits (Qiagen Inc., Valencia, CA) following manufacturer protocols.

**Table 1:** Sampling locations and sample sizes for microsatellite and mtDNA sequencing analyses. Map codes refer to those highlighted in Fig. 1

Sample	N (Microsats)	N (Sequencing)	Map Code	Latitude	Longitude
Dease Arm	80	36	1	66°30′	120°22′
Keith Arm	95	29	2	65°10′	123°29′
Keith Arm-Manitou	45	na	3	64°58′	122°06′
Keith Arm-Russell	74	na	4	65°28′	122°51′
McTavish Arm	85	23	5	66°11′	117°58′
McVicar Arm	97	19	6	65°24′	120°22′
Smith Arm	79	28	7	66°18′	124°18′
Sandy Lake	41	22	8	67°48′	132°15′

Eleven microsatellite loci were amplified in three multiplex reactions (Appendix A1). Amplified microsatellite fragments were analyzed using an automated sequencer (ABI 3130×l Genetic Analyzer; Applied Biosystems, Foster City, CA) with the LIZ 600 size standard. All genotypes were scored using GeneMapper (ver. 4.0, Applied Biosystems) software.

Mitochondrial DNA variation was assessed by sequencing the control region (d-loop) following modifications from Power et al. (#b^202^). Briefly, the left domain of the d-loop region was amplified with primers tPro2 (Brunner et al. 2001) and ARCH1 (5′-CCY TGT TAG ATT TYT TCG CTT GC-3′; Alekseyev et al. 2009). Sequencing of the target amplification product was accomplished with primer tPro2 using the Applied Biosystems Big Dye Terminator v3.1 Terminator Cycle Sequencing kit (Applied Biosystems). Sequencing reaction products were run on an Applied Biosystems 3130×l Genetic Analyzer. Sequences were aligned to the most common lake trout haplotype (Snam1) using Seqscape vers. 2.5 (Applied Biosystems).

### Analysis of Microsatellite Genetic Variation

For each sampling location, MICROCHECKER (ver. 2.2.3, van Oosterhout et al. 2004) was used to test for genotyping errors in the form of null alleles, large allele dropout and allele scoring errors. For each locus within each sample, microsatellite descriptive statistics, including number of alleles (N_A_), expected (H_E,_ Nei's unbiased gene diversity) and observed (H_O_) heterozygosities and the fixation index (F_IS_) were calculated in FSTAT (ver. 2.9.2.3, Goudet 2002). Accounting for differences in sample size, the program HP-RARE (Kalinowski 2005) was used to calculate allelic richness (A_R_, 100 genes from each sample) and private allelic richness (PA_R,_ 100 genes from each sample).

Deviations from Hardy–Weinberg equilibrium (HWE) for each locus–population combination and linkage disequilibrium (LD) for each locus–population-pair combination were detected using GENEPOP (ver. 4.0, Rousset 2008) employing exact tests and a Monte Carlo (MC) algorithm (Guo and Thompson 1992) to estimate *P*-values. The significance of simultaneous comparisons was initially compared with a nominal alpha of 0.05 and then to an adjusted alpha following the false discovery rate procedure (Narum 2006).

Given that these systems have likely been colonized relatively recently (perhaps within the last 5000 years, (Lindsey and McPhail 1986)), we tested for bottlenecks, which are often associated with recent post-glacial colonization and range expansion (Bernatchez and Wilson 1998). We used the program BOTTLENECK (ver. 1.2.02, Piry et al. 1999) for comparing H_E_ to that expected under mutation-drift equilibrium (H_EQ_). As allele number declines more rapidly than heterozygosity during bottlenecks, recently bottlenecked populations should exhibit heterozygosity excess relative to that expected given the observed number of alleles. Both the step-wise mutation (SMM) and the two-phase mutation (TPM) models (95% single-step mutations and 12% variance of multi-step mutations, Piry et al. 1999) were assumed in making calculations using Wilcoxon signed-rank tests (Luikart and Cornuet 1998) with 1000 iterations.

### Analysis of microsatellite population structure

Genotypic differentiation among sample pairs, across all loci combined, was estimated in GENEPOP using log-likelihood G-based exact tests (Goudet et al. 1996) with default values for dememorization, number of batches, and iterations per batch. To determine whether allele size (vs. state) is a more appropriate descriptor of differentiation in this system (in which case R_ST,_ instead of F_ST_, would be a better estimator of differentiation), we employed the allele size randomization procedure (10,000 permutations) as implemented in SPAGeDi 1.1 (Hardy and Vekemans 2002). Results indicating that observed R_ST_ is significantly larger than that generated through the permutation process (pR_ST_) would suggest that stepwise mutations are important in the current structuring of differentiation (Hardy et al. 2003). In this assessment, F_ST_ appears to provide a more appropriate measure of differentiation (see Results) and therefore this estimator was used instead of R_ST_. Global F_ST_, specifically Weir and Cockerham's (1984) θ, was calculated using FSTAT and differences in this measure among all samples, and only those from GBL were tested using 10,000 permutations. Pairwise F_ST_ among all samples was compared in ARLEQUIN (ver. 3.1, Excoffier et al. 2005) and significance was assessed using 10,000 permutations.

To resolve the influence of geographic distance between sampling locations on the genetic structuring of lake trout populations, isolation-by-distance analyses (IBD) were performed using Rousset's (1997) framework based on F_ST_/(1−F_ST_). Patterns of IBD were tested for all samples combined and then for those including GBL only by examining the correlation between fluvial distance and genetic distance (F_ST_/(1−F_ST_)). The significance of these relationships was tested using Mantel (1967) tests in R (ver. 2.12.1) employing 10,000 permutations.

Several analyses were performed to visualize population structure among lake trout samples. First, using GENETIX (ver. 4.02, Belkhir et al. 2004), we performed a factorial correspondence analysis (FCA) to visually assess genetic variation at the individual level in multi-dimensional space. Second, Bayesian model-based clustering implemented in STRUCTURE (ver. 2.3.1, Pritchard et al. 2000) was used to estimate the most likely number of distinct genetic groups (clusters (*K*)) given our multi-locus genotype data without any a priori designation of populations. We performed STRUCTURE analyses for two data sets: (1) one consisting of all samples; and (2) one containing only samples from GBL. We employed an admixture model and assumed that allele frequencies were correlated. Given initial evidence of weak population structure in this system (see Results), STRUCTURE analyses were performed using location (LOCPRIOR) information (see Hubisz et al. 2009). The number of clusters (*K*) was identified using 20 replicate runs for each possible value of *K* (1–10) using a burn-in of 10,000 followed by 10,000 MCMC steps (longer trial runs produced the same results). We report, for the number of clusters, both the posterior probability of the data (ln P[D]) and the post hoc Δ*K* statistic of Evanno et al. (2005).

### Assessment of dispersal and contemporary and historical gene flow

Contemporary dispersal among sampling locations was first assessed employing the “detection of first generation migrants” option implemented in the program GENECLASS2 (Piry et al. 2004). This option identifies immigrant individuals in the current generation (*F*_0_ individuals) as those that display genotypic disequilibrium relative to their sampled population (Paetkau et al. 2004). For migrant detection in GENECLASS2, we used the test statistic *Lh/Lmax* and analyses were performed using the Bayesian approach of Rannala and Mountain (1997) and the re-sampling procedure of Paetkau et al. (2004). We employed a probability threshold of *α* = 0.05, below which any individual was assigned as a migrant. We also plotted the genotype likelihoods and calculated the test statistic *D*_*LR*_ (mean genotype likelihood ratios, (Paetkau et al. 2004)) as a method for assessing power for detecting migrants given our data set.

Second, we used the Bayesian approach as implemented in the program BAYESASS (ver. 3.0, Wilson and Rannala 2003) to estimate contemporary gene flow (recent migration rates, *m*_BA_) among sampling locations. Similar to the detection of *F*_0_ migrants using GENECLASS2, immigrants in BAYESASS are identified as individuals that display genotypic disequilibrium relative to the population from where they were sampled. BAYESASS, however, estimates recent migration within the past one to three generations (Wilson and Rannala 2003). Each run was performed using 1 × 10^7^ iterations, a burn-in of 1 × 10^6^ and a sampling frequency of 100. As recommended in the user manual, mixing parameters (allele frequencies (a), inbreeding (f), and migration rate (m)) were all varied until acceptance rates were ∼30%. Once mixing rates were optimized, ten independent runs, with varying random seed numbers, were performed and results are presented as averages of these runs. We first assessed recent migration among arms within GBL and then between GBL and Sandy Lake. The program TRACER (ver. 1.5, Rambaut and Drummond 2007) was used as a method to qualitatively assess Markov chain Monte Carlo (MCMC) convergence.

Finally, historical gene flow (*M*_MIG_) among arms within GBL and between Sandy Lake and GBL was estimated using the maximum-likelihood approach in MIGRATE (ver. 3.0.3, Beerli and Felsenstein 2001; Beerli 2008). This program uses coalescent theory and MCMC to concurrently estimate historical migration rates (M) scaled by mutation (=*m*/*μ*) and population size (Θ = 4*N*_*e*_μ. Beerli 2006). MIGRATE analyses were performed assuming an infinite allele model (IAM) following the default settings (10 short chains with a sampling increment of 100 where 500 genealogies are sampled; three long chains with a sampling increment of 1000 where 5000 genealogies are sampled) from a subsample of 50 individuals per population or 100 per lake when comparing GBL to Sandy Lake. We discarded (burn-in) 10,000 genealogies at the beginning of each chain and employed an adaptive heating scheme with four temperatures (1.0, 1.5, 2.5, and 3.0). Maximum-likelihood estimates and credible intervals are reported and, as with the BAYESASS analysis, MIGRATE results were averaged over five independent runs.

### Analysis of mitochondrial (Sequence) genetic variation and population structure

MEGA ver. 5.0 (Tamura et al. 2011) was used to test for the most appropriate model of nucleotide substitution. Both Bayesian Information Criterion (BIC) and Akaike Information Criterion (corrected, AICc) suggested the Tamura 3-parameter model (Tamura 1992) as the most appropriate model of nucleotide substitution and therefore this model was used in all subsequent analyses that required prior substitution information. Descriptive statistics such as the frequency of mtDNA haplotypes, haplotype diversity (*h*) and nucleotide diversity (П) were calculated using ARLEQUIN ver. 3.01 (Excoffier et al. 2005). Tajima's D, a test for deviations from neutral expectations (Tajima 1989), and pairwise *F*_ST_ comparisons of haplotype frequencies between sampling locations were also calculated in Arlequin with significance of these estimates tested by employing 10,000 permutations. Finally, the program TCS ver. 1.20 (Clement et al. 2000) was used to construct a haplotype network based on the statistical parsimony method of Templeton et al. (1992) to visualize the evolutionary relationships among haplotypes.

## Results

### Genetic variation at microsatellite loci

The locus SnaMSU6 was identified to contain a null allele at all but one sampling location, and therefore, was removed from all subsequent analyses. Microsatellite variation in lake trout was therefore assayed at 10 loci across 596 individuals from eight sampling locations (Appendix A2). Overall, allelic variation was high ranging from four (Smm21) to 27 (SnaMSU12) alleles per locus with an average of 14.8 across all loci (Appendix A2). Expected heterozygosity (H_E_) ranged from 0.34 (Smm21) to 0.86 (Sco202 and SnaMSU12) and averaged 0.70 across all loci. Conformation to Hardy–Weinberg equilibrium (HWE) was rejected in 14 of a possible 80 population–locus comparisons (*P* < 0.05). All of these involved deficits of heterozygotes and deviations from HWE appear to be distributed randomly among loci and sampling locations. Subsequent to adjustments of alpha based on the false discovery rate procedure, eight comparisons involved a significant deviation (*P* < 0.00979) from HWE. Significant linkage disequilibrium was detected in 28 of 360 tests (*P* < 0.05), but after employing the false discovery rate procedure, it was detected in only nine comparisons (*P* < 0.0073), four of which involved the Manitou sample from Keith Arm.

There was no evidence of recent bottlenecks in any of the samples under either of the models of mutation employed (Table 2). Although each sample contained loci with higher than expected heterozygosity given the observed number of alleles, no significant deviations from equilibrium were detected (all *P* > 0.05).

**Table 2:** Wilcoxon sign-rank tests for heterozygosity excess among eight sampling locations for *Salvelinus namaycush* under stepwise (SMM) and two-phase models (TPM) of mutation. He / Hd represents the ratio of the number of loci with a heterozygosity excess to the number with a heterozygosity deficiency where *P* represents the statistical significance of any deviation from equilibrium (non-bottleneck) expectations (≤1:1)

	TPM		SMM	
		
Population	He/Hd	*P*	He/Hd	*P*
Dease Arm	4/6	0.95801	3/7	0.99316
Keith Arm	3/7	0.83887	3/7	0.99512
Keith Arm-Manitou	2/8	0.95801	2/8	0.98779
Keith Arm-Russell	3/7	0.88379	3/7	0.99512
McTavish Arm	2/8	0.98389	1/9	0.99854
McVicar Arm	4/6	0.88379	3/7	0.99072
Smith Arm	2/8	0.99512	1/9	0.99902
Sandy Lake	1/9	0.99902	1/9	0.99902

### Genetic differentiation at microsatellite loci

G-based exact tests revealed that 21 of 28 pairwise comparisons among samples were significantly differentiated from each other (*P* < 0.05, Table 3). Subsequent to the false discovery rate adjustments, this decreased to 12 pairwise comparisons (*P* < 0.0127) including all comparisons with Sandy Lake and three that included the McVicar Arm of GBL.

**Table 3:** Results of log-likelihood, G-based exact tests for genetic differentiation among pairs of populations (above diagonal) where values are significant (underlined) subsequent to adjustments of alpha based on the false discovery rate procedure (adjusted alpha of 0.0127). Pairwise *F*_ST_ (θ) comparisons among all pairs of populations (below diagonal) where values are significant (underlined) subsequent to adjustments of alpha based on the false discovery rate procedure (adjusted alpha of 0.0127)

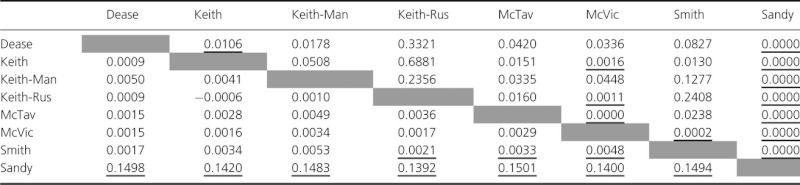

**Figure 2:**
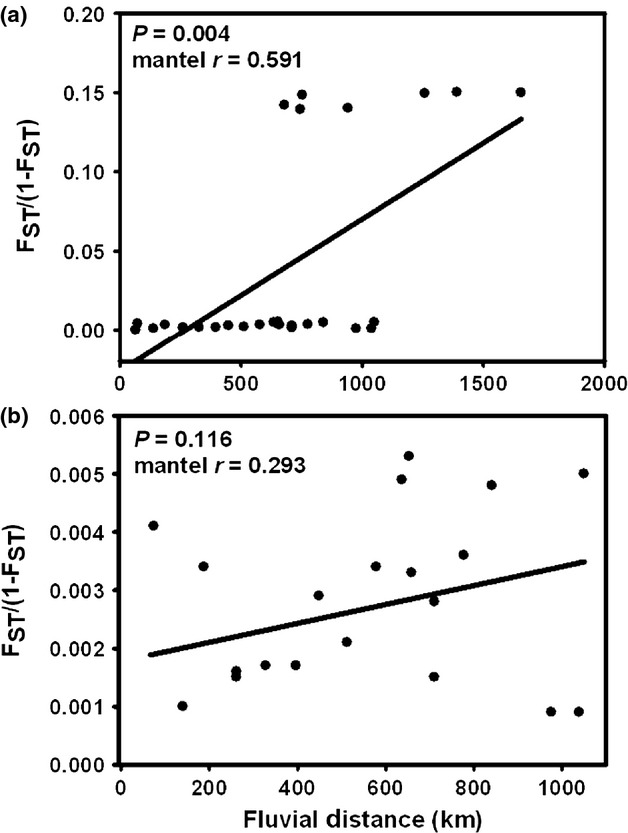
Isolation-by-distance patterns over all sampling locations (a), and then when including only those from Great Bear Lake (b). *F*_ST_ as defined by Weir and Cockerham (1984).

Results from the allele size permutation tests in SPAGeDi suggested that only 12 of a possible 280 comparisons showed evidence that allele size significantly contributed to differentiation, and therefore *F*_ST_ (θ) (and not *R*_ST_) was considered in further analyses. Overall, low, but significant, genetic differentiation was found (global *F*_ST_ = 0.025, 95% C.I. = 0.016–0.034, *P* < 0.05) and this was highly inflated by comparisons which included Sandy Lake. Among the GBL only samples, global *F*_ST_ was still significant, but tenfold lower (0.002, 95% C.I. = 0.001–0.003). Among sampling locations, pairwise *F*_ST_ ranged from −0.006 between two locations within Keith Arm of GBL to 0.150 between Sandy Lake and Smith Arm of GBL (Table 3). Within GBL, the largest differentiation was observed between Smith Arm and Keith Arm-Manitou (*F*_ST_ = 0.0053). Thirteen of these 28 pairwise comparisons were significant (*P* < 0.05), but only 10 pairwise comparisons remained significant after employing the false discovery rate procedure (*P* < 0.0127). Pairwise comparisons that included Sandy Lake were always significant and produced the highest *F*_ST_ estimates (Table 3). Significant IBD across the study area was observed when all samples were included in the analysis (Mantel *r* = 0.591, *P* = 0.004, Fig 2a). When only GBL samples were included, however, the correlation between genetic (F_ST_/(1−F_ST_)) and geographic distance was much less pronounced and was non-significant (Mantel *r* = 0.293, *P* = 0.116, Fig 2b).

**Figure 3:**
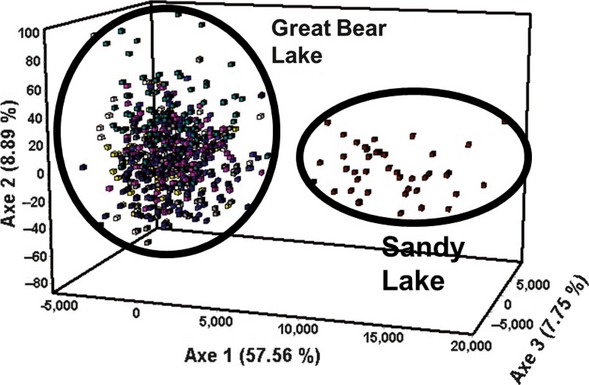
Factorial correspondence analysis highlighting the genetic discontinuity between Sandy and Great Bear lakes.

The FCA (Fig. 3) clearly shows the genetic discontinuity between GBL and Sandy Lake samples, while also highlighting the genetic homogeneity among GBL samples. Great Bear Lake populations, or individuals within GBL populations, were clearly differentiated from their Sandy Lake counterparts while little structure was resolved among samples from different locations within GBL (Fig. 3).

**Figure 4:**
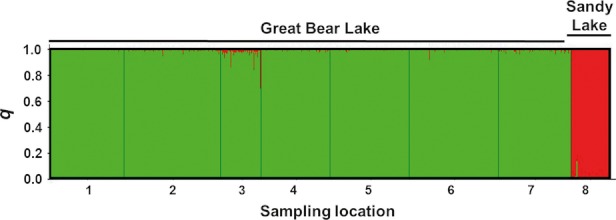
Results of the STRUCTURE (at K = 2, see results) analysis showing the proportion of the genome (*q,* admixture coefficient) assigned to one of the two most likely inferred clusters (red or green). Each column represents a different individual. Sampling locations refer to those outlined in Table 1 and Fig. 1.

Similarly, results from Bayesian clustering implemented in STRUCTURE clearly suggested that two genetic populations/clusters (*K*) were present and this was the case regardless of the test statistic employed (i.e., Δ*K* (=143.02) and lnP[D] (=−21,057.24), Table 4, Fig. 4). Bar plots of the admixture coefficient (*q*) clearly show the genetic discontinuity between GBL and Sandy Lake while highlighting the genetic homogeneity within the GBL samples. Indeed, when STRUCTURE was performed solely on samples from GBL, the number of inferred genetic groups was one (lnP[D] = −19861.13, data not shown, note: computationally it is not possible to calculate Δ*K* for an inferred cluster (*K*) of one).

**Table 4:** Statistics from Bayesian clustering implemented in STRUCTURE (Pritchard et al. 2000) across all sampling locations calculated from 20 iterations. Shown are the mean and standard deviations (SD) of the log-likelihood values (LnP[D]) for different hypothesized numbers of genetic populations (*K*). Also shown is the mean value of *ΔK*, the ad hoc statistic of Evanno et al. (2005) used to summarize the second-order rate of change in LnP(D). The underlined value of *K* = 2 represents the most likely number of genetic groups indicated by both test statistics. NA = not applicable given that *ΔK* cannot be calculated for these values of *K*

K	MeanlnP(D)	SDlnP[D]	ΔK
1	−21373.74	0.58	NA
2	−**20837.24**	4.78	**143.02**
3	−20984.75	90.64	0.64
4	−21074.57	154.60	0.58
5	−21254.73	196.83	0.46
6	−21344.47	285.13	0.24
7	−21501.61	233.38	0.34
8	−21739.21	492.76	0.25
9	−21851.49	595.42	0.26
10	−21810.54	440.59	NA

### Estimates of contemporary dispersal and historical gene flow

Twelve of 596 individuals were identified as *F*_0_ migrants using the “detection of first generation migrants” option in GENECLASS2 (Table 5). There appeared to be no consistent pattern of dispersal (immigration) among arms within GBL (i.e., migrants were distributed among all arms within this system). Given the lack of detectable structure, however, and our analysis of the genotype likelihoods and low values of the test statistic *D*_*LR*_ (Appendix A3a), power to detect migrants among arms of GBL may be hampered. No migrants were detected between GBL and Sandy Lake and plots of genotype likelihood values and values of the test statistic *D*_*LR*_ between GBL and Sandy Lake indicated sufficient power for detecting migrants between these two systems (Appendix A3b).

**Table 5:** Results of the assessment for detecting first-generation migrants performed using GENECLASS2 showing the number of individual migrants (*P* < 0.01) detected per sampling location. Results are based on the *Lh/Lmax* statistic

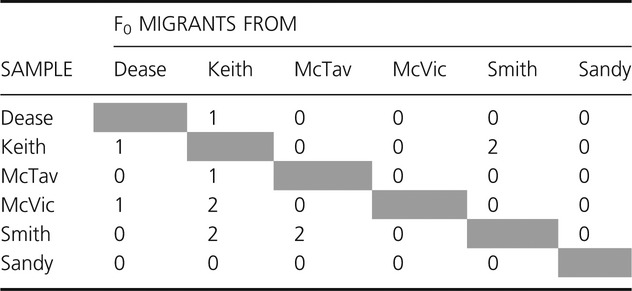

**Figure 5:**
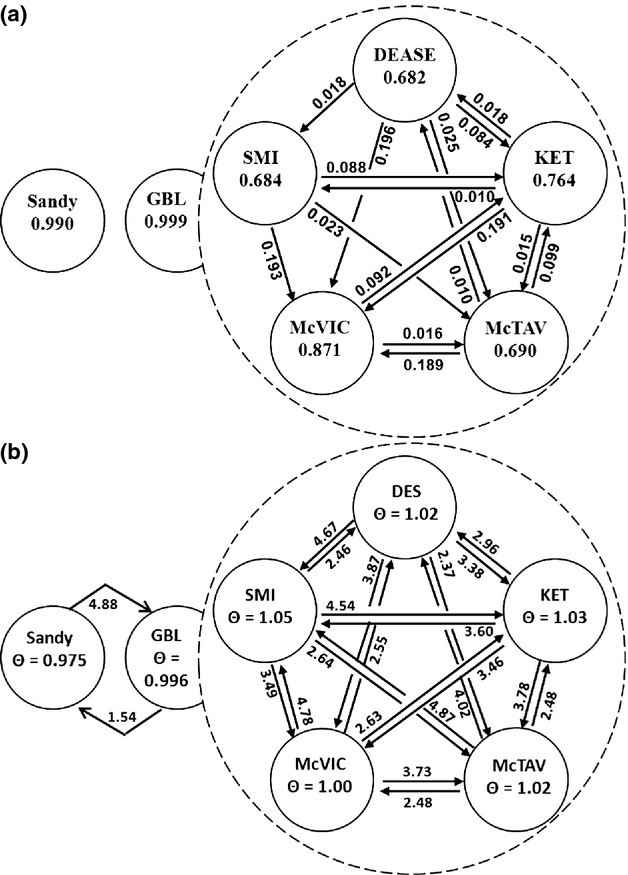
Mean estimates and inferred directions of contemporary gene flow (a, assessed using BAYESASS) and historical gene flow (b, assessed using MIGRATE) for lake trout between sample arms within GBL (enclosed within the dashed circle) and among the GBL and Sandy Lake systems. Contemporary results are averaged over 10 independent runs and only migration rates (*m*, the proportion of the population that migrated in the direction show by the arrow per generation) estimates greater than 0.01 are shown. Historical gene flow estimates were averaged over five independent runs and also shown for each sampling location is the value of theta (Θ = 4*N*_E_*μ*). The 95% C.I.'s of each mean value for both analyses are shown in Appendix A5.

Bayesian estimates of contemporary migration among arms within GBL were variable (Fig. 5a, Appendix A4). For example, estimates of migration were as high as 0.196 (migration from Dease Arm into McVicar Arm) and as low as <0.01 (among several arms within the lake). In general, however, and in contrast to the results from GENECLASS, it appears that migration into McVicar Arm was high and asymmetrical compared with migration among other arms within GBL. Contemporary gene flow inferred from BAYESASS was essentially non-existent between GBL and Sandy Lake (Fig. 5a). Trace plots and marginal density plots created using the program TRACER suggest that convergence of MCMC runs was not a concern and that contemporary estimates of gene flow are likely reliable (Appendix A5).

Finally, long-term migration rates (scaled by mutation rate; *M* = *m*/*μ*) estimated using the likelihood approach in MIGRATE tended to be variable and asymmetrical among arms within GBL (Fig. 5b, Appendix A4). Although historical migration appears to be prevalent within GBL, no consistent pattern could be resolved (Fig. 5b). For example, estimates of long-term migration were as low as 2.46 (from Smith Arm into Dease Arm) and as high as 4.87 (from McTavish Arm into Smith Arm). Long-term estimates of migration between Sandy Lake and GBL were within the range reported for those among arms within GBL. Gene flow, however, was consistently asymmetrical with migration prominently in the direction of Sandy Lake to GBL (Appendix A4).

### mtDNA variation and population structure

**Figure 6:**
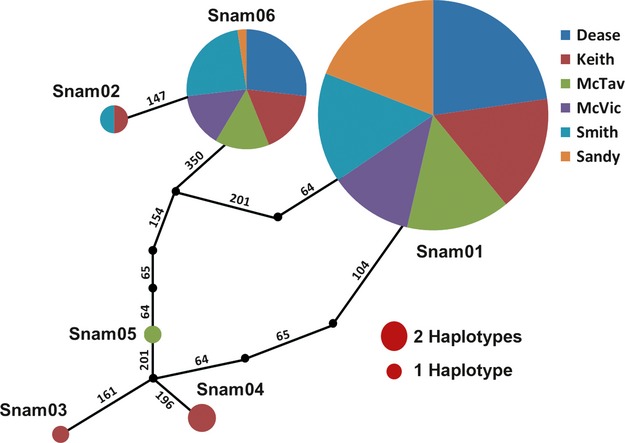
Unrooted haplotype network of the mtDNA control region showing the geographic distribution of haplotypes produced using TCS (Clement et al. 2000). Haplotypes are colored according to geographic localities shown in the legend. The sizes of the haplotypes reflect the number of specimens sharing the same haplotype (see scale in the lower right corner).

The left domain of the mtDNA control region was sequenced for 158 lake trout from six sampling locations (five within GBL and one from Sandy Lake). Of the 468 base pairs sequenced, eight variable positions were resolved (Table 6) resulting in six unique haplotypes (Table 7, Fig. 6); all of which were deposited into GenBank (accession numbers: JQ772460–JQ772465). Haplotype *Snam01* was most common among lake trout samples (*N* = 110) and this haplotype was relatively well represented within each arm of GBL and within Sandy Lake (Table 7, Fig. 6). Haplotype *Snam06* was also relatively common (*N* = 41), was found throughout GBL and was represented by one sample in Sandy Lake (Table 7). The other four haplotypes were represented by only one (haplotypes *Snam02* and *Snam03*) or two samples (haplotypes *Snam04* and *Snam05*). Polymorphism within each sample was consistent with neutral expectations (Tajima's *D* = −1.162–1.814, *P* > 0.05, Table 7). Estimates of mtDNA variability (nucleotide and haplotype diversity) were relatively similar among arms within GBL; however, variability within Sandy Lake was much lower (Table 7). Overall, nucleotide diversity ranged from 0.008 within Sandy Lake to 0.0043 within Keith Arm of GBL. Haplotype diversity was moderate in GBL (*h* = 0.4365−0.569) and much lower in Sandy Lake (*h* = 0.0909, Table 7).

**Table 6:** Alignment of the polymorphic sites seen in the left domain of the mtDNA control region. All variation is relative to the most common lake trout haplotype (Snam1). All new lake trout haplotype sequences are deposited in Genbank under accession numbers JQ772460–JQ772465. Filled squares represent similarity to the reference haplotype (Snam01)

	Nucleotide Position							
	
Haplotype	64	65	147	154	161	196	201	350
(Snam01)	C	A	A	**–**	A	C	T	A
(Snam02)	**–**	**–**	**▪**	A	**▪**	**▪**	C	**▪**
(Snam03)	**–**	**–**	**▪**	A	G	**▪**	**▪**	**▪**
(Snam04)	**–**	**–**	**▪**	**▪**	**▪**	**▪**	**▪**	**▪**
(Snam05)	T	**▪**	G	**▪**	**▪**	**▪**	C	G
(Snam06)	T	**▪**	**▪**	**▪**	**▪**	**▪**	C	G

**Table 7:** mtDNA (d-loop) sequencing statistics and number of haplotypes per sampling location. Statistics shown per location include the number of haplotypes, nucleotide diversity (π), haplotype diversity (*h*), and Tajima's D. Also shown are the GenBank accession numbers for lake trout haplotypes resolved in this study (Snam01-06)

Haplotype	Dease	Keith	McTav	McVic	Smith	Sandy	GenBank Accession #
Snam01	25	18	16	13	17	21	JQ772460
Snam02	0	0	1	0	0	0	JQ772461
Snam03	0	1	0	0	0	0	JQ772462
Snam04	0	2	0	0	0	0	JQ772463
Snam05	0	1	0	0	1	0	JQ772464
Snam06	11	7	6	6	10	1	JQ772465
Total Number of Haplotypes	36	29	23	19	28	22	
Total Unique Haplotypes	2	5	3	2	3	2	
Nucleotide Diversity	0.0028	0.0043	0.0032	0.0029	0.0033	0.0008	
Haplotype Diversity	0.4365	0.5690	0.4664	0.4561	0.5212	0.0909	
Tajima's D	1.8142	−0.1406	1.2937	1.6159	1.3431	−1.1624	

Similar to the analysis of microsatellite population structure, analysis of mtDNA sequences revealed no significant population structure among GBL locations as evidenced by the *F*_ST_ values (all *P* ≥ 0.05, Table 8). Significant structure, however, was observed when comparing Sandy Lake with any sampling location within GBL (*P* < 0.05). Genetic homogeneity across our study area was also evidenced by the parsimonious haplotype network (Fig. 6). The two most common haplotypes (*Snam01* and *Snam06*) were virtually equally represented in all arms of GBL and were also found in Sandy Lake. The remaining four haplotypes, separated by varying numbers of mutations, were quite rare and were only represented in a total of six individuals.

**Table 8:** Pairwise *F*_ST_ comparisons of haplotype frequencies between sampling locations

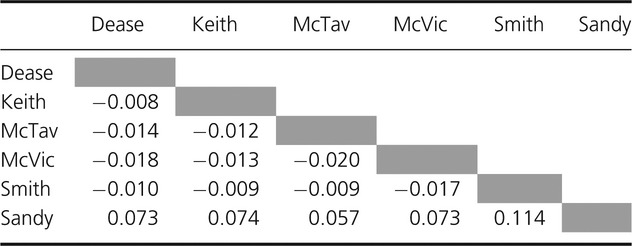

## Discussion

In this study, we assessed population structure of lake trout in one of Canada's largest, relatively undisturbed freshwater ecosystems. Using a combination of molecular markers, this study is one of the first contributions to understanding genetic structure in Arctic populations of this species. Overall, our results suggest a lack of genetic structure among samples of lake trout from GBL (overall mtDNA/microsatellite *F*_ST_ ∼ −0.013/0.002) while showing considerable differentiation among samples from GBL and Sandy Lake, which is separated from GBL by distances comparable to those between the various GBL sampling sites (overall mtDNA/microsatellite *F*_ST_ ∼ 0.079/0.15 between lakes).

Assessments of population structure in lake trout are quite rare (Hendry and Stearns 2004) and contemporary population structure resolved among GBL lake trout samples using microsatellite DNA variation is much weaker than what has been reported in other large-lake genetic population structure assessments for this species. For example, in Lake Superior lake trout, Piller et al. (2005) found an *F*_ST_ of 0.025 between two localities in this system separated by more than 350 km. Similar to our study, these authors also resolved comparable values of differentiation (*F*_ST_ ∼ 0.111) between lake trout from Lake Superior and an out-group population from another nearby lake, Lewis Lake, which harbors populations of lake trout descended from a northern Lake Michigan population (Page et al. 2003). Burnham-Curtis (1993), however, through an assessment of mtDNA RFLP, observed weak intra-lake structure among lake trout from Lake Superior (sequence divergence estimates: 0.0005–0.0159). In general, estimates of contemporary differentiation among lake trout from the Laurentian Great Lakes should be interpreted with caution as these values are likely obscured given the long history of extensive hatchery supplementation programs applied in these systems (Krueger and Ihssen 1995; Page et al. 2004). In the only other comparable system (with respect to size and lack of habitat perturbation) in which the lake trout population structure has been assessed, the Atlin-Tagish Lake system in British Columbia, Northrup et al. (2010) resolved three genetic sub-populations and found more comparable, albeit higher, overall levels of population structure (*F*_ST_ range: 0.005–0.027). The overall higher levels, and more pronounced structure, among lake trout in this system may be the result of differing sampling strategies and landscape/lake features. Northrup et al. (2010) sampled individuals on known spawning reefs in Tagish Lake (although Atlin lake samples were obtained from fixed-lake surveys), but in GBL, little information is available with respect to spawning locations. Furthermore, Atlin and Tagish lakes are connected only by the Atlin River, a feature that may restrict gene flow within this system. In contrast, within GBL, there are no obvious physical restrictions to gene flow and therefore population subdivision is expected to be lower among these continuously distributed populations. Indeed, the level of *F*_ST_ resolved for GBL lake trout using microsatellite DNA is among one of the lowest reported in salmonid fishes (Hendry and Stearns 2004).

In general, however, intra-lake assessments of population structure in salmonids are relatively rare (Hendry and Stearns 2004). Studies that are available typically show that there is regularly a lack of differentiation among populations/samples for lake-spawning species (Dynes et al. 1999; Stewart et al. 2003), as is the case in this study. Alternatively, populations spawning in tributaries to lakes tend to be much more genetically structured (Fraser et al. 2004; D'Amelio and Wilson 2008; Stott et al. 2010). This result may be attributed to a higher propensity for natal homing in river spawning fishes, a phenomenon that quite prevalent in salmonids (Dittman and Quinn 1996; Quinn 2005; Neville et al. 2006). Natal fidelity, however, has also been documented in lake-spawning salmonids (Stewart et al. 2003).

Our analyses of mtDNA (d-loop) also revealed relatively little lake-wide variation, although some range-wide geographic structure was evident (i.e., between Sandy Lake and GBL). The lack of intra-lake differentiation among populations of salmonid fishes, as measured using mtDNA markers, appears to be a common phenomenon (Bernatchez and Dodson 1991; Wilson et al. 1996; Turgeon and Bernatchez 2001b; Alekseyev et al. 2009; Witt et al. 2011) including, specifically, populations of lake trout (Wilson and Hebert 1996, 1998; Piller et al. 2005). In GBL, Wilson and Hebert (1998) employed RFLP analysis of mtDNA and resolved very little intra-lake structure among lake trout. These authors only found two haplotypes in this system, although the sample size was not large enough to accurately assess intra-lake structure (*N* = 5). Lack of intra-lake structure is common in systems such as GBL that appear to be “evolutionarily young” (Bernatchez and Wilson 1998). For example, many freshwater Nearctic systems have been post-glacially colonized within the last 10,000 years (or even more recently, Lindsey and McPhail 1986; Rempel and Smith, 1998), and in most systems, by one glacial lineage (Hansen et al. 1999; Stamford and Taylor 2004). As such, sufficient time has often not yet passed, and thus if effective population sizes have been high historically, mutation and genetic drift are negligible in promoting differentiation and population structure among these founding populations (Harris and Taylor 2010b). These effects are especially pronounced in species like lake trout that have relatively long generation times (approximately 15 years, K.L. Howland, unpublished data, and see below). Indeed, the relatively weak patterns of IBD for our samples collected solely within GBL suggest that these populations have yet to reach migration-drift equilibrium (Hutchison and Templeton 1999). Combined, both nuclear and mtDNA assessed in GBL lake trout, strongly suggest a general lack of neutral genetic differentiation in this system.

Several factors may help to explain the lack of detectable genetic structure within GBL. First, contemporary gene flow among GBL lake trout populations may be pervasive. This would indicate that homing to natal spawning areas may be imprecise, resulting in high rates of straying and subsequent gene flow among populations. Gene flow acts as a homogenizing force among populations (Slatkin 1985, 1987), and if contemporary gene flow among GBL lake trout populations is widespread, then a lack of differentiation may not be surprising. Unfortunately, relatively little is known regarding straying rates in this species, but a few studies suggest that straying may not be uncommon based on long-distance movement in this species (Rybicki 1990; Adlerstein et al. 2007). Physical tagging results, however, suggest that lake trout populations of GBL tend to move relatively little within this system. Over a nine year assessment, Johnson (1975b) found that most lake trout were recaptured very close to tagging locations although a few fish had apparently moved >40 km with a maximum recorded distance of 71.6 km. Substantial movements in lake trout, however, have been reported from the Great Lakes. For example, in Lake Superior, Eschmeyer et al. (1953) reported movements of lake trout approaching 400 km. Long-distance movement (>160 km) of lake trout has also been reported in Great Slave Lake (Keleher 1963), a system more similar to that in this study. In general, however, lake trout appear to move relatively little (Keleher 1963; Johnson 1975b; Schmalz et al. 2002). High levels of gene flow have also been reported among populations of the congeneric brook trout (*Salvelinus fontinalis*). For instance, D'Amelio and Wilson (2008) used molecular assays to infer high levels of gene flow among populations of brook trout from Lake Huron. Fraser et al. (2004) also found movement of brook trout among spawning tributaries, where long-distance dispersal appeared to be asymmetrical. Alternatively, other studies have resolved a strong propensity for spawning fidelity in this species (Mucha and Mackereth 2008). Our estimates of contemporary migration (assessed using BAYESASS) indicate that there are likely occasional movements of lake trout among some arms within the GBL system. Spawning locations within GBL are not well known, but it is possible that spawning areas are not discretely located, which might contribute to a lack of population structure if fish sampled from different arms congregate in a small number of continuously distributed spawning reefs.

Second, the lack of genetic differentiation among the samples from GBL may be the result of factors such as a strong signal of historical gene flow and the retention of ancestral allelic polymorphism, large historical effective population sizes (*N*_E_), or combinations of these influences. Historical gene flow and the retention of ancestral polymorphisms have been implicated in the lack of differentiation and obscured contemporary population structure in a variety of other northern and Arctic salmonids (e.g., Harris and Taylor 2010b; Pilgrim et al. 2012). Pronounced historical gene flow in this study is evidenced by the lack of differentiation at mtDNA between Sandy and GBL lakes (sharing of common haplotypes between these systems), although these two systems are clearly isolated from each other based on microsatellite DNA analysis. Furthermore, estimates of long-term gene flow produced from MIGRATE analyses provide evidence for historical gene flow between Sandy Lake and GBL and among arms within GBL, a result that may potentially mask or obscure contemporary structure in this system (e.g., Taylor et al. 2011; Pilgrim et al. 2012). This may be especially true for systems recently colonized post-glacially by founding populations with extremely large historical effective sizes where drift is essentially negligible. Indeed, we were unable to detect any recent bottlenecks suggesting that population sizes have not been reduced drastically as this system was colonized. For example, in other salmonids, estimates of historical *N*_E_ have approached 500,000 in northern, post-glacially colonized systems, which typically were much larger in physical area early in deglaciation (Stamford and Taylor 2005). If founding GBL lake trout population sizes were similarly large, the lack of differentiation in this system may not be surprising especially given the relatively recent time since colonization and the longer generation time for this species. It is probable that this system may have been colonized by lake trout as recently as ∼ 5000 years ago (McPhail and Lindsey, 1986) and with a generation time of 15 years in GBL (Scott and Crossman #b^203^), GBL lake trout populations have had a maximum of approximately 330 generations to diverge from one another. This may not be a sufficient amount of time (Estoup and Angers #b^200^) for the evolution of pronounced genetic differentiation in GBL populations of lake trout. We used our MIGRATE-derived estimates of theta to calculate *N*_E_ within GBL and the estimate of ∼330 generations since the founding of this system to calculate an expected *F*_ST_ under isolation following the expression *F*_ST_ = 1−(1−1/(2N_E_))^*t*^ (Nei and Chakravarti 1977) where *t* is the elapsed time (in generations) the populations diverged from a common ancestor. This resulted in an expected global *F*_ST_ within GBL under complete isolation of 0.0066, more than three times as high as our empirical estimate of 0.002. This result is consistent with the idea that gene flow among populations has occurred, and likely continues to occur, since the founding of GBL. In fact the lack of detectable differentiation in this system (i.e., apparent panmixia), it is likely that both contemporary (ongoing dispersal and gene flow) and historical (historical gene flow and large founding population sizes) factors contribute to this lack of neutral genetic structure. Further physical tagging studies to assess movements within the lake and molecular assessments will be important for distinguishing among these alternative (or complementary) hypotheses more fully.

The lack of detectable neutral genetic structure in lake trout from GBL does not mean, however, there is no genetic structure within this large and complex system. For instance, different morphological types of lake trout have been reported from this system (Blackie et al. 2003; Alfonso 2004). If these differences reflect, at least in part, inherited variation as is commonly observed in salmonids (e.g., Adams and Huntingford 2004; Keeley et al. #b^201^), then this needs to be accounted for in a broader assessment of population structure and its significance in GBL.

## Conclusions

This study represents the first genetic assessment of population structure within one of Canada's largest and most northern lake ecosystems and one of the few assessments of lake trout outside of the Laurentian Great Lakes. Assessing nuclear and mtDNA variation, we documented extremely low levels of intra-lake population structure (i.e., among locations within GBL), which we did not anticipate given the size and complexity of GBL: our study reports some of the lowest values of differentiation among salmonid populations across such a large geographic scale. We also observed genetic differences between Sandy Lake and GBL highlighting significant inter-lake structure; results that are consistent with the only other assessment of lake trout genetic structure among pristine, northern populations (see Northrup et al. 2010). Overall, our study highlights the potential importance of both contemporary and historical factors in shaping the population structure in Arctic systems colonized within the last 10,000 years and we suggest that these forces are acting in concert in GBL resulting in a state of apparent panmixia. Given that in GBL, the confounding effects of anthropogenic impacts on population structure and genetic diversity are essentially negligible, the genetic structure resolved in this study may provide a more realistic view of genetic structure among recently colonized, pristine populations of long-lived salmonids in relatively unstructured physical habitats.

## Supporting Information

**Appendix A1.** Polymerase Chain Reaction (PCR^a^) Information.

**Appendix A2.** Basic descriptive statistics for 10 microsatellite loci for the 8 sampling locations assessed showing the average number of alleles per locus (N_A_), observed (H_O_) and expected (H_E_) heterozygosities, F_IS_ inbreeding coefficient, allelic richness (A_R_), and private allelic richness (PA_R_).

**Appendix A3.** Shown are typical log-likelihood plots for assignment of first generation migrants among, for example, Great Bear Lake (GBL) and Sandy Lake (A) and among two arms within GBL (B). Qualitatively, power to detect migrants appears very high when comparing between systems, but is hampered when inferring first generation migrants among arms within GBL. Quantitatively, *D*_*LR*_ a test statistic used to assess power of assignment tests (Paetkau et al. [Bibr b71]) was high between GBL and Sandy Lake (*D*_*LR*_ *˜* 10 when comparing between systems) and very low when comparing arms within GBL (*D*_*LR*_ < 3 between any two arms within GBL) indicating high power of assignment between systems, but not among arms within GBL. Paetkau et al. ([Bibr b71]) state “a value of *DLR* in excess of 5 was always associated with near maximum power to distinguish immigrants and residents, while a value below 3 was associated with power of less than 0.5”.

**Appendix A4.** Mean estimates and 95% C.I.'s for of long-term migration calculated using the program MIGRATE. Shown are estimates of migration among arms in GBL (A) and migration between Sandy Lake and GBL (B) averaged over five independent runs.

**Appendix A5.** Trace plots and marginal density plots of MCMC runs from BAYESASS. Shown is the trace plot and marginal density plot for estimates of contemporary gene flow among arms within GBL (A and B, respectively) and the trace plot and marginal density plot for estimates of contemporary gene flow between Sandy Lake and GBL systems (C and D, respectively). All results are averaged over 10 independent BAYESASS runs.
