# Supplementary material for: Microsatellite and mtDNA analysis of lake trout, Salvelinus namaycush, from Great Bear Lake, Northwest Territories: impacts of historical and contemporary evolutionary forces on Arctic ecosystems
Source: Ecol Evol. 2013 Jan 10;3(1):145–61. doi: 10.1002/ece3.439 (PMC3568850; doi:10.1002/ece3.439)

**SUPPORTING INFORMATION**

Appendix 1. Polymerase Chain Reaction (PCR^a^) Information

| ^a^ PCR Protocol: 1 x 95^o^C for 5 min; 35 x 95^o^C for 30 sec, 55^o^C for 30 sec, 72^o^C for 45 sec; 1 x 72^o^C for 30 min |
| --- |
| ^b^ reverse primer includes a custom tailed (a 7 base sequence added onto the 5’ end, see Brownstein et. al. 1996) |
| ^c^ forward primer labelled with flourescent dye |
| ^d^AmpliTaq Gold® DNA Polymerase with Gold Buffer and MgCl_2_ solution from Applied Biosystems |

Appendix 2. Basic descriptive statistics for ten microsatellite loci for the 8 sampling locations assessed showing the average number of alleles per locus (N_A_), observed (H_O_) and expected (H_E_) heterozygosities, F_IS_ inbreeding coefficient, allelic richness (A_R_) and private allelic richness (PA_R_).

|  |  |  |  |  |  |  |  |  |  |  |  |
| --- | --- | --- | --- | --- | --- | --- | --- | --- | --- | --- | --- |
| **Dease Arm** | **OtsG83** | **Smm21** | **Smm17** | **Sco215** | **OMM1105** | **OtsG23** | **SSOSL4** | **Smm22** | **Sco202** | **SnaMSU12** | **Average** |
| **N_A_** | 12 | 4 | 13 | 8 | 11 | 11 | 3 | 20 | 12 | 18 | **11.20** |
| **H_E_** | 0.87 | 0.41 | 0.83 | 0.75 | 0.70 | 0.81 | 0.29 | 0.93 | 0.87 | 0.92 | **0.74** |
| **H_O_** | 0.85 | 0.40 | 0.85 | 0.69 | 0.67 | 0.76 | 0.23 | 0.88 | 0.70 | 0.89 | **0.69** |
| **F_IS_** | 0.02 | 0.03 | -0.02 | 0.08 | 0.05 | 0.06 | 0.20 | 0.05 | 0.19 | 0.04 | **0.07** |
| **A_R_^*^** | 11.44 | 3.49 | 12.29 | 7.28 | 10.42 | 9.87 | 3.00 | 18.26 | 11.81 | 17.13 | **10.50** |
| **PA_R_*** | 0.05 | 0.00 | 0.96 | 0.77 | 0.00 | 0.18 | 0.00 | 0.23 | 0.97 | 1.66 | **0.48** |
|  |  |  |  |  |  |  |  |  |  |  |  |
| **Keith Arm** | **OtsG83** | **Smm21** | **Smm17** | **Sco215** | **OMM110** | **OtsG23** | **SSOSL4** | **Smm22** | **Sco202** | **SnaMSU12** | **Average** |
| **N_A_** | 11 | 3 | 14 | 7 | 12 | 10 | 3 | 21 | 14 | 22 | **11.70** |
| **H_E_** | 0.86 | 0.35 | 0.87 | 0.71 | 0.72 | 0.80 | 0.24 | 0.93 | 0.90 | 0.90 | **0.73** |
| **H_O_** | 0.85 | 0.36 | 0.87 | 0.64 | 0.67 | 0.70 | 0.22 | 0.91 | 0.84 | 0.84 | **0.69** |
| **F_IS_** | 0.02 | -0.05 | 0.01 | 0.10 | 0.08 | 0.12 | 0.09 | 0.02 | 0.06 | 0.06 | **0.05** |
| **A_R_^*^** | 10.65 | 2.48 | 12.38 | 6.44 | 10.15 | 8.87 | 3.00 | 18.73 | 13.70 | 18.15 | **10.46** |
| **PA_R_*** | 0.00 | 0.00 | 0.93 | 0.06 | 0.00 | 0.03 | 0.00 | 0.20 | 0.00 | 0.99 | **0.22** |
|  |  |  |  |  |  |  |  |  |  |  |  |
| **Keith Arm- Man.** | **OtsG83** | **Smm21** | **Smm17** | **Sco215** | **OMM110** | **OtsG23** | **SSOSL4** | **Smm22** | **Sco202** | **SnaMSU12** | **Average** |
| **N_A_** | 9 | 4 | 11 | 5 | 9 | 9 | 3 | 19 | 11 | 14 | **9.40** |
| **H_E_** | 0.81 | 0.27 | 0.81 | 0.79 | 0.72 | 0.79 | 0.10 | 0.93 | 0.89 | 0.88 | **0.70** |
| **H_O_** | 0.71 | 0.24 | 0.69 | 0.73 | 0.67 | 0.73 | 0.11 | 0.79 | 0.80 | 0.76 | **0.62** |
| **F_IS_** | 0.13 | 0.12 | 0.15 | 0.08 | 0.07 | 0.08 | -0.03 | 0.15 | 0.11 | 0.14 | **0.10** |
| **A_R_^*^** | 9.00 | 4.00 | 11.00 | 5.00 | 9.00 | 9.00 | 3.00 | 19.00 | 11.00 | 14.00 | **9.40** |
| **PA_R_*** | 0.00 | 0.00 | 0.00 | 0.00 | 0.00 | 0.03 | 0.00 | 0.51 | 0.00 | 1.00 | **0.15** |
|  |  |  |  |  |  |  |  |  |  |  |  |
| **Keith Arm - Russ.** | **OtsG83** | **Smm21** | **Smm17** | **Sco215** | **OMM110** | **OtsG23** | **SSOSL4** | **Smm22** | **Sco202** | **SnaMSU12** | **Average** |
| **N_A_** | 12 | 3 | 11 | 6 | 12 | 11 | 4 | 18 | 14 | 19 | **11.00** |
| **H_E_** | 0.85 | 0.37 | 0.82 | 0.75 | 0.77 | 0.76 | 0.28 | 0.92 | 0.90 | 0.91 | **0.73** |
| **H_O_** | 0.81 | 0.41 | 0.88 | 0.63 | 0.76 | 0.77 | 0.25 | 0.88 | 0.88 | 0.87 | **0.71** |
| **F_IS_** | 0.05 | -0.11 | -0.07 | 0.17 | 0.01 | -0.01 | 0.11 | 0.05 | 0.02 | 0.04 | **0.02** |
| **A_R_^*^** | 11.33 | 2.89 | 10.67 | 5.99 | 10.95 | 10.02 | 3.67 | 17.16 | 13.77 | 18.03 | **10.45** |
| **PA_R_*** | 0.06 | 0.00 | 0.18 | 0.00 | 0.67 | 0.00 | 0.67 | 0.00 | 0.01 | 0.81 | **0.24** |
|  |  |  |  |  |  |  |  |  |  |  |  |
| **McTavish Arm** | **OtsG83** | **Smm21** | **Smm17** | **Sco215** | **OMM110** | **OtsG23** | **SSOSL4** | **Smm22** | **Sco202** | **SnaMSU12** | **Average** |
| **N_A_** | 12 | 3 | 12 | 6 | 11 | 12 | 3 | 21 | 13 | 19 | **11.20** |
| **H_E_** | 0.88 | 0.37 | 0.83 | 0.71 | 0.75 | 0.80 | 0.12 | 0.91 | 0.87 | 0.90 | **0.71** |
| **H_O_** | 0.88 | 0.27 | 0.79 | 0.63 | 0.78 | 0.79 | 0.13 | 0.82 | 0.82 | 0.86 | **0.68** |
| **F_IS_** | 0.00 | 0.26 | 0.04 | 0.12 | -0.04 | 0.01 | -0.05 | 0.10 | 0.06 | 0.05 | **0.05** |
| **A_R_^*^** | 11.52 | 2.97 | 10.98 | 5.82 | 10.51 | 9.86 | 2.98 | 18.49 | 12.45 | 17.07 | **10.27** |
| **PA_R_*** | 0.58 | 0.00 | 0.32 | 0.00 | 0.00 | 0.14 | 0.00 | 0.35 | 0.00 | 0.91 | **0.23** |
|  |  |  |  |  |  |  |  |  |  |  |  |
| **McVicar Arm** | **OtsG83** | **Smm21** | **Smm17** | **Sco215** | **OMM110** | **OtsG23** | **SSOSL4** | **Smm22** | **Sco202** | **SnaMSU12** | **Average** |
| **N_A_** | 12 | 3 | 13 | 6 | 13 | 12 | 3 | 19 | 13 | 17 | **11.10** |
| **H_E_** | 0.87 | 0.42 | 0.86 | 0.76 | 0.75 | 0.78 | 0.20 | 0.91 | 0.88 | 0.88 | **0.73** |
| **H_O_** | 0.84 | 0.45 | 0.81 | 0.72 | 0.72 | 0.78 | 0.21 | 0.90 | 0.89 | 0.86 | **0.72** |
| **F_IS_** | 0.04 | -0.07 | 0.05 | 0.05 | 0.04 | -0.01 | -0.03 | 0.01 | -0.01 | 0.03 | **0.01** |
| **A_R_^*^** | 10.99 | 2.99 | 11.30 | 5.99 | 11.14 | 10.16 | 3.00 | 16.74 | 12.32 | 15.92 | **10.06** |
| **PA_R_*** | 0.10 | 0.00 | 0.12 | 0.00 | 0.52 | 0.08 | 0.00 | 0.11 | 0.00 | 0.41 | **0.13** |
|  |  |  |  |  |  |  |  |  |  |  |  |
| **Smith Arm** | **OtsG83** | **Smm21** | **Smm17** | **Sco215** | **OMM110** | **OtsG23** | **SSOSL4** | **Smm22** | **Sco202** | **SnaMSU12** | **Average** |
| **N_A_** | 11 | 3 | 11 | 8 | 13 | 13 | 3 | 22 | 14 | 17 | **11.50** |
| **H_E_** | 0.86 | 0.34 | 0.83 | 0.68 | 0.68 | 0.81 | 0.16 | 0.92 | 0.87 | 0.91 | **0.70** |
| **H_O_** | 0.67 | 0.32 | 0.78 | 0.69 | 0.65 | 0.73 | 0.14 | 0.90 | 0.85 | 0.91 | **0.66** |
| **F_IS_** | 0.22 | 0.07 | 0.05 | -0.01 | 0.05 | 0.09 | 0.10 | 0.02 | 0.02 | 0.00 | **0.06** |
| **A_R_^*^** | 10.48 | 2.95 | 10.58 | 7.06 | 10.89 | 11.46 | 2.99 | 19.38 | 13.54 | 16.16 | **10.55** |
| **PA_R_*** | 0.00 | 0.00 | 0.06 | 0.77 | 1.27 | 0.69 | 0.00 | 0.93 | 0.00 | 0.10 | **0.38** |
|  |  |  |  |  |  |  |  |  |  |  |  |
| **Sandy Lake** | **OtsG83** | **Smm21** | **Smm17** | **Sco215** | **OMM110** | **OtsG23** | **SSOSL4** | **Smm22** | **Sco202** | **SnaMSU12** | **Average** |
| **N_A_** | 9 | 2 | 5 | 4 | 5 | 7 | 3 | 14 | 7 | 6 | **6.20** |
| **H_E_** | 0.61 | 0.22 | 0.69 | 0.44 | 0.54 | 0.73 | 0.23 | 0.85 | 0.73 | 0.58 | **0.56** |
| **H_O_** | 0.53 | 0.25 | 0.50 | 0.34 | 0.47 | 0.70 | 0.19 | 0.84 | 0.49 | 0.49 | **0.48** |
| **F_IS_** | 0.14 | -0.13 | 0.27 | 0.22 | 0.12 | 0.04 | 0.14 | 0.01 | 0.34 | 0.16 | **0.13** |
| **A_R_^*^** | 9.00 | 2.00 | 5.00 | 4.00 | 5.00 | 7.00 | 3.00 | 14.00 | 7.00 | 6.00 | **6.20** |
| **PA_R_*** | 0.00 | 0.00 | 0.00 | 0.00 | 0.00 | 1.49 | 1.00 | 0.00 | 0.00 | 0.00 | **0.25** |
|  |  |  |  |  |  |  |  |  |  |  |  |
| **All Locations** | **OtsG83** | **Smm21** | **Smm17** | **Sco215** | **OMM110** | **OtsG23** | **SSOSL4** | **Smm22** | **Sco202** | **SnaMSU12** | **Average** |
| **N_A_** | **13** | **4** | **17** | **9** | **16** | **17** | **5** | **25** | **15** | **27** | **14.80** |
| **H_E_** | **0.83** | **0.34** | **0.82** | **0.70** | **0.70** | **0.78** | **0.20** | **0.91** | **0.86** | **0.86** | **0.70** |
| **H_O_** | **0.77** | **0.34** | **0.77** | **0.63** | **0.67** | **0.75** | **0.18** | **0.86** | **0.78** | **0.81** | **0.66** |
| **F_IS_** | **0.08** | **0.02** | **0.06** | **0.10** | **0.05** | **0.05** | **0.07** | **0.05** | **0.10** | **0.06** | **0.06** |
| **A_R_^*^** | **10.55** | **2.97** | **10.52** | **5.95** | **9.76** | **9.53** | **3.08** | **17.72** | **11.95** | **15.31** | **9.73** |
| **PA_R_*** | **0.10** | **0.00** | **0.32** | **0.20** | **0.31** | **0.33** | **0.21** | **0.29** | **0.12** | **0.73** | **0.26** |

*AR and PAR with both calculated by sampling 100 genes.

Appendix 3. Shown are typical log-likelihood plots for assignment of first generation migrants among, for example, GBL and Sandy Lake (A) and among two arms within GBL (B). Qualitatively, power to detect migrants appears very high when comparing between systems, but is hampered when inferring first generation migrants among arms within GBL. Quantitatively, *D_LR_* a test statistic used to assess power of assignment tests (Paetkau *et al.,* 2004) was high between GBL and Sandy Lake (*D_LR_ ~* 10 when comparing between systems) and very low when comparing arms within GBL (*D_LR_* < 3 between any two arms within GBL) indicating high power of assignment between systems but not among arms within GBL. Paetkau et al., (2004) state “a value of *DLR* in excess of 5 was always associated with near maximum power to distinguish immigrants and residents, while a value below 3 was associated with power of less than 0.5”.


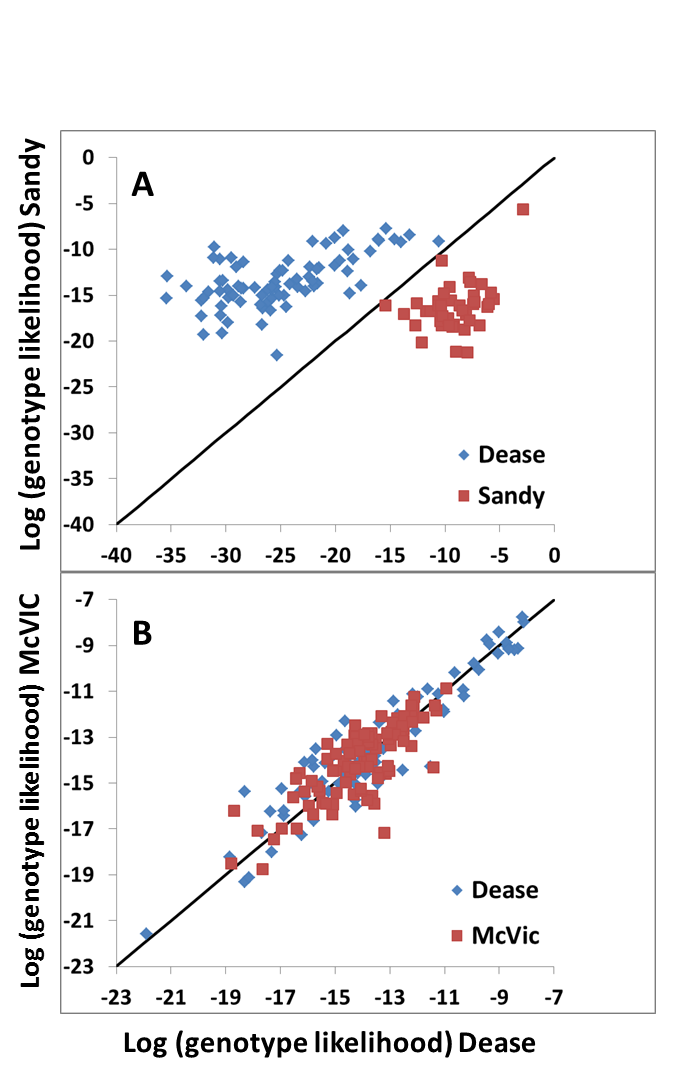


Appendix 4. Mean estimates and 95% C.I.’s for of long term migration calculated using the program MIGRATE. Shown are estimates of migration among arms in GBL (A) and migration between Sandy Lake and GBL (B) averaged over five independent runs.

Appendix 5. Trace plots and marginal density plots of MCMC runs from BAYESASS. Shown is the trace plot and marginal density plot for estimates of contemporary gene flow among arms within GBL (A and B respectively) and the trace plot and marginal density plot for estimates of contemporary gene flow between Sandy Lake and GBL systems (C and D respectively). All results are averaged over 10 independent BAYESASS runs.


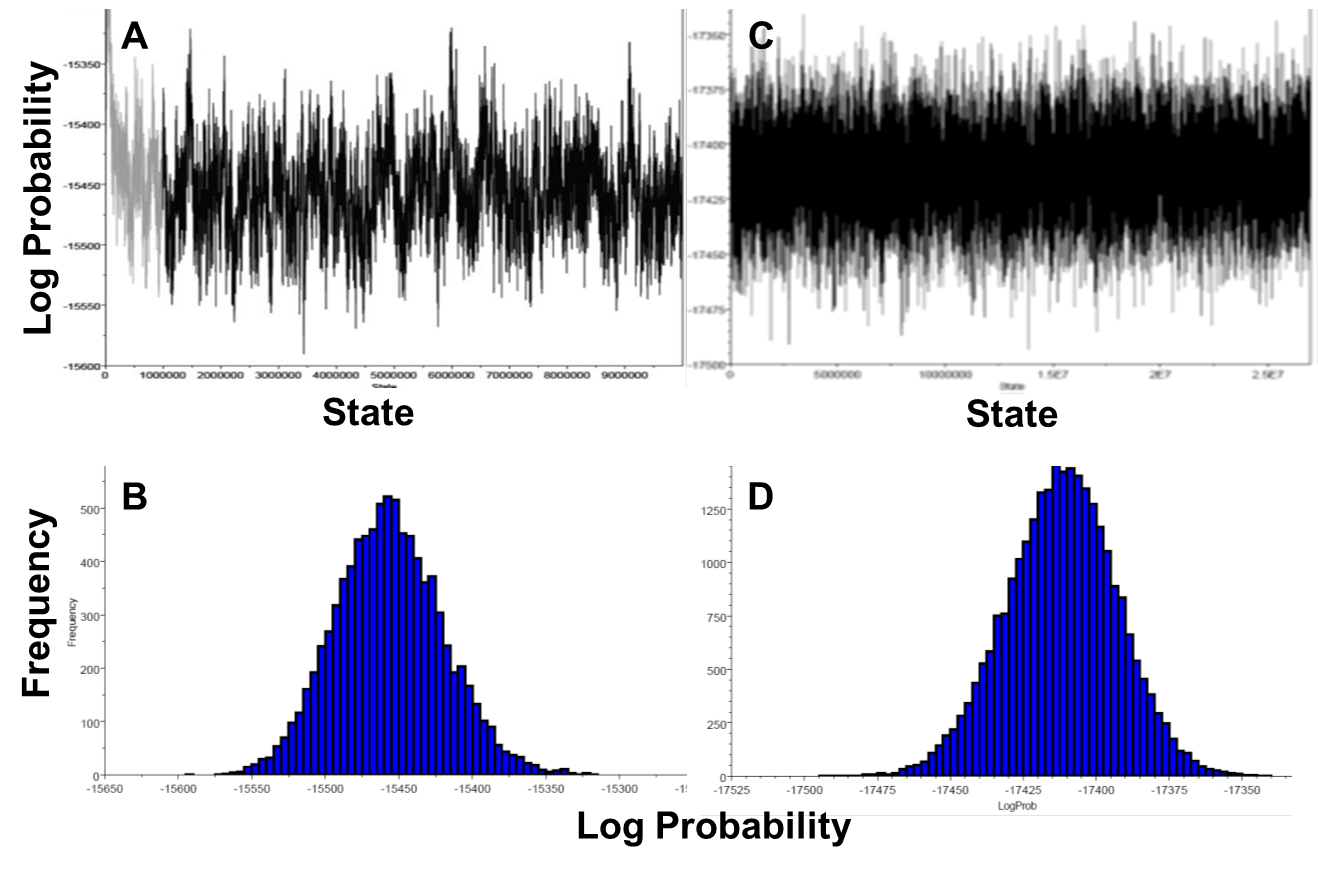

Supplement: Supplementary file 1 [file ece30003-0145-SD1.docx]
